# Unilateral Vocal Cord Paralysis of a Great Jewish Opera Singer

**DOI:** 10.5041/RMMJ.10322

**Published:** 2018-01-29

**Authors:** Irit Duek, Jacob T. Cohen, Ziv Gil

**Affiliations:** 1 Department of Otolaryngology Head and Neck Surgery, The Head and Neck Center, Rambam Health Care Campus, Haifa, Israel; 2 Rappaport Institute of Medicine and Research, The Technion–Israel Institute of Technology, Haifa, Israel

**Keywords:** George London, postviral neuropathy, unilateral vocal cord palsy

## Abstract

George London was one of the most compelling vocal artists of the early twentieth century. At the age of 47, the great bass-baritone retired from singing. It has been suggested that the premature ending of his operatic career was due to unilateral vocal cord palsy (UVCP). When London retired, the common belief was that this UVCP was caused by viral hepatitis, although there is no evidence to support such an etiology. London’s medical records eliminate the possible etiology of a neck neoplasm, and the long period of time between a heart attack he experienced and his diagnosis of UVCP makes a cardiovascular etiology an unlikely causative factor. London’s relatively young age, the diagnosis of laryngitis prior to his UVCP, and the course of his disease indicate that the underlying cause of the termination of his singing career was post-viral neuropathy. This paper describes the clinical evidence related to London’s vocal cord function and explores the possible causes for his UVCP, which apparently led to his early retirement.

## INTRODUCTION

**Figure 1:**
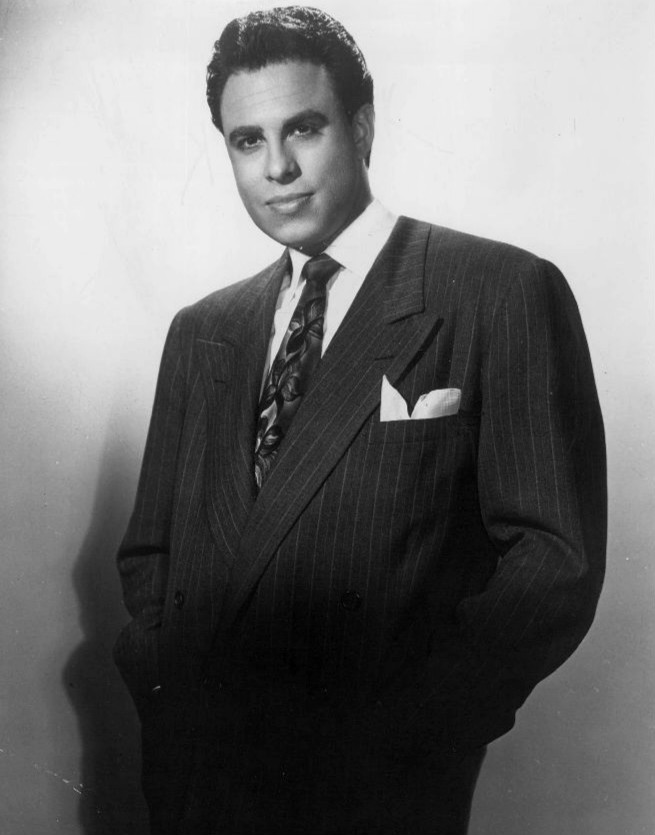
George London in 1951.

George London (1920–1985) was one of the greatest bass-baritone singers of all times (Figure 1). Born George Burnstein to Russian Jewish parents in Montreal, he began his vocal training at the age of 15, after his family moved to Los Angeles. He made his operatic debut in 1946 as Doctor Grenvil in Verdi’s *La Traviata* at the Hollywood Bowl. In 1949, he joined the Vienna State Opera. At the age of 30 London appeared at the Glyndebourne Festival. He made his Bayreuth Festspiele debut a year later as King Amfortas, in Wagner’s last opera *Parsifal*. This was the first appearance of a North American singer in Bayreuth, home of the Richard Wagner Opera Festival, which became a cultural symbol for the Third Reich. In 1951, London joined the Metropolitan Opera; in 1960, he was the first North American singer to perform in the Bolshoi Theater in Moscow. Unfortunately, London began to suffer from abrupt deterioration of his voice at the age of 41 and retired from singing several years later to embrace a conducting career at the National Opera Institute.^1^–^7^

## CASE PRESENTATION

According to the biographical writings of London’s wife,^4^,^5^ London’s first vocal disturbance occurred during a tour with the Metropolitan Opera in April 1960. She wrote: “his voice cracked on the F-sharp, the high note, toward the end of his third-act aria, ‘Vedro mentr’io sospiro’.”^5^^(p. 156)^ However, he experienced no other vocal difficulties for the rest of the tour.

In the winter of 1961, London suffered from an upper respiratory tract infection. He was examined by Dr Leo Reckford, an otolaryngologist who took care of most of the Metropolitan Opera’s artists at the time. His physical examination raised suspicions of right vocal cord palsy; however, a clear diagnosis could not be made since London’s throat was inflamed. At an examination a few days later, after the inflammation and swelling resolved, Dr Reckford recorded impaired movement of London’s right vocal cord with complete vocal cord adduction at phonation. The diagnosis initially had no influence on London’s singing. At several consecutive examinations by different throat specialists, it seemed that the right cord, though impaired in its movement, still moved slightly.

In 1964, London underwent surgery to straighten his nasal septum, following a recommendation from a German doctor (Dr Zimmermann), with no change in his vocal condition. As the years went by, it became obvious that London’s right vocal cord was not moving, and a diagnosis of permanent right vocal cord paralysis was made. One year after being diagnosed with complete vocal cord paralysis, London’s voice was gradually deteriorating, and compared to his normal voice (http://bit.ly/2zUorJt) it became weaker and more hoarse (http://bit.ly/2zCG3IZ). Laryngeal examination in 1966 revealed right vocal cord atrophy. No remedy was offered by the different specialists he saw, until he went to Los Angeles to consult with Dr Henry Rubin. In the 1960s, Dr Henry Rubin was practicing in treating selected dysphonias with vocal cord injections of synthetic materials—Teflon and silicone.^8^–^12^ He proposed a silicone injection to London’s paralyzed right vocal cord. The next morning London received the injection, with immediate excellent results. Two days later he could talk almost normally. According to Mrs London, “he was still a little hoarse, but both the speaking and the singing voice retained the same quality and exact tone as before the paralysis.” 5^(p. 169)^ Although his voice improved following the injection, by the end of 1966 London decided to retire from singing—despite the improvement, the quality of his singing voice would not suffice. Over the years, as the silicone was resorbed, London’s speaking voice also deteriorated, and he subsequently received two additional Teflon injections.

## POSSIBLE CAUSES OF LONDON’S VOCAL PARALYSIS

Unilateral vocal cord palsy (UVCP) is frequently asymptomatic though it may lead to dysphonia as well as dysphagia. Associated symptoms include hoarseness, voice fatigue, cough, aspiration, and swallowing difficulties. The degree of UVCP depends on the position of paralyzed vocal cord and on muscle tone. Vocal cord paralysis signifies vocal fold immobility that is restricted secondary to mechanical fixation or neuropathy, with several possible causes (Table 1).^13^–^29^ Mechanical fixation may result from arytenoid dislocation, edema or inflammation of the glottis, or neoplastic invasion. Neurogenic immobility can be caused by a disease process of any part of the vagus nerve from its origin in the medullary nucleus ambiguous or its supranuclear tracts in the brain, to the recurrent laryngeal nerve.^13^–^22^ Brain tumors, from brainstem to jugular foramen, strokes, and demyelinating diseases are rarer etiologies. Recurrent laryngeal nerve paralysis can be also caused by non-neoplastic space-occupying lesions including aortic aneurysm, mitral stenosis, and mediastinal cysts. Penetrating or blunt trauma can also cause vocal cord dysfunction. Systemic causes of recurrent laryngeal paralysis include lead poisoning, bacterial infections (such as diphtheria), or viral illness. More than 85% of UVCP^13^–^22^ has three main causes: (1) nerve injury during surgery (of thyroid or mediastinum), (2) neoplastic invasion along the path of the recurrent laryngeal nerve, and (3) an inflammatory process usually due to a viral infection.

**Table 1:** Possible Underlying Causes of UVCP and Their Incidence.

Reference	# of Patients	Surgery (%)	Neoplasm (%)	Idiopathic (%)	Trauma (%)	Others (%)
Gupta et al.^14^	112	10.71	34.82	13.39	9.82	31.26
Nerurkar et al.^15^	85	44.71	14.12	16.47	11.76	12.94
Chen et al.^16^	259	39.38	31.27	10.81	7.72	10.82
Rosenthal et al.^17^	827	46.3	13.5	17.6	2.2	20.4
Ozbal Koc et al.^18^	92	50	9.8	31.5	1.1	7.6
Ko et al.^19^	161	48.4	11.8	21.7	7.4	10.7
Guha et al.^21^	50	54.9	7.8	31.4	5.9	0
Parnell & Brandenburg^23^	86	23	36	11	2.3	27.7
Titche^24^	128	9.4	39	2.3	10.9	38.4
Shei et al.^25^	283	22	39	11	14.3	13.7
Terris et al.^26^	84	34.5	40.5	10.7	8.3	6
Benninger et al.^27^	280	24	25	20	18	13
Ramadan et al.^28^	98	29.6	31.6	16.3	7.1	15.4
Yumoto et al.^29^	422	42.7	22.4	17.4	2.1	15.4

London’s vocal cord dysfunction was evident when he was 41 years old, with complete paralysis developing over the following three years. The next sections examine the possible causes of London’s vocal cord dysfunction.

### Hepatitis and Other Viral Infections

Mrs London suggested that her husband’s UVCP was due to a hepatitis infection following a vitamin B injection with a needle that “was not new and did not seem too clean,”^5^^(p. 154)^ while on tour in Israel in 1959. Upon his return to the United States, London was diagnosed and hospitalized for viral hepatitis (most probably hepatitis A virus, though the precise type is unknown). Mrs London firmly believed the hepatitis was the only possible explanation for the UVCP, as she wrote: “recently, it has been discovered that hepatitis sometimes attacks a nerve; this was probably the only valid explanation.” (p. 163)^5^ However, there is no evidence in the literature that any form of viral hepatitis can cause mononeuropathy presenting as isolated UVCP. Hepatitis infection is found to be associated with various systemic diseases such as systemic rheumatoid vasculitis and systemic lupus erythematosus, in which vagal neuropathy and laryngeal involvement are reported and documented,^30^–^34^ however there are no clues suggesting that London had suffered from such diseases. In addition, 1959–1960 were documented as very successful performing years.^4^,^5^

Other viral infections such as Epstein–Barr virus,^35^–^38^ cytomegalovirus,^39^ herpes simplex virus (HSV),^40^–^42^ varicella zoster virus,^43^,^44^ West Nile virus,^45^ and other agents causing upper respiratory tract infection^40^,^46^ have been reported as possible causes of vocal cord palsy. However, since they are not blood-borne pathogens it is unlikely that they led to London’s UVCP.

### Vitamin B12 Deficiency or Toxicity

Data retrieved from London’s biographies suggested that he frequently received vitamin B injections although no such deficiency was documented (according to his wife, “he believed it gave him added energy”^5^^(p. 154)^). There is no evidence in the literature that vitamin B12 hyper-vitaminosis could cause UVCP. At the most, high serum cobalamin levels can sometimes be paradoxically accompanied by signs of vitamin B12 deficiency. Vitamin B12 deficiency has a wide variety of neurological symptoms and signs. However, cranial neuropathies other than optic neuropathy have been rarely reported. Hoarseness with vocal cord paralysis, myelopathy, and peripheral neuropathy have been described as unusual neurological manifestations of B12 deficiency.^47^,^48^ However, in London’s case, there is no description of symptoms that may be associated with vitamin B12 deficiency or toxicity, thus this etiology is less likely to be the cause of his UVCP.

### Cardiovascular Disease

Ortner’s (cardiovocal) syndrome is a vocal fold paralysis resulting from compression or stretching of the recurrent laryngeal nerve in its intra-thoracic trajectory as a consequence of cardiovascular changes (such as aortic arch aneurysm).^49^ The left recurrent laryngeal nerve, with its longer course around the aortic arch, is more frequently involved. In London’s case, the right vocal cord was paralyzed, lowering the possibility of a cardiac abnormality leading to his UVCP. The vascular cause can be ruled out as a cause of London’s UVCP since 16 years passed from his initial diagnosis to his first experienced myocardial infarction (in 1977, at the age of 57). During that time he was asymptomatic for cardiovascular issues.

### Neoplasm

Malignant neoplasm has been reported as the most common cause of extra-laryngeal vocal cord paralysis.^13^ Neck neoplasms including those of the thyroid, esophagus, and upper lungs are often complicated by vocal cord paralysis.^16^,^19^,^50^ However, London underwent neck exploration in 1964, to reveal whether some structures were pressing on the recurrent laryngeal nerve. According to his medical records, the surgeons found no evidence for cervical neoplasm.^4^,^5^

### Idiopathic Vocal Cord Paralysis

Most published reports indicate that 10%–20% of UVCP cases have no clear etiology^13^–^22^,^51^ and postulate a viral cause.^41^,^42^

It is theoretically possible that London’s UVCP developed long before the upper respiratory infection in 1961, leading to an inadvertent finding upon indirect laryngoscopy. However, idiopathic UVCP is reported to have a 50% spontaneous recovery rate.^13^ Since London’s UVCP progressively worsened, an idiopathic cause may be ruled out.

### Postviral Vagal Neuropathy

Cranial nerves are known to be affected by inflammatory neuropathic processes that are possibly viral,^46^ causing isolated nerve injuries resulting in sensorimotor dysfunction (transient cranial mononeuropathy^46^). Postviral vagal neuropathy (PVVN) has similarities with other postviral neuropathic disorders such as glossopharyngeal neuralgia and Bell’s palsy.^40^,^46^ Upper respiratory infections due to influenza A and other viruses can give rise to multiple cranial nerve palsies, including ophthalmoplegia, facial weakness, or dysphagia with paralysis of the soft palate and vocal cords.^46^ Recurrent laryngeal neuritis may develop in relation to a HSV infection.^40^–^42^

There are two proposed mechanisms whereby viral infection may cause neuritis or neuropathy: (1) direct infection and inflammation of a nerve,^52^ and (2) induction of a non-specific inflammatory response, secondarily involving a nerve. Various inflammatory mediators may cause indiscriminate damage to the nerve through demyelination and axonal loss or may lead to the production of cross-reacting antibodies, which may subsequently damage the nerve. This acute inflammatory response may also temporarily slow nerve conduction.^40^

While suffering from an acute upper respiratory tract infection in 1961, London was diagnosed with a laryngeal infection and presumably impaired movement of his right vocal cord. A few days later, after the laryngeal swelling and inflammation subsided, his vocal cords were in a central position, but the right vocal cord seemed paretic. Subsequent examinations indicated that his right cord was moving, but, as the years passed, it became evident that his right vocal cord was permanently paralyzed. Five years after London’s first diagnosis, laryngeal examination revealed right vocal cord atrophy, which most likely occurred due to impaired innervation of the vocalis muscle.

There is a clear association between the proven diagnosis of laryngeal infection and right vocal cord paresis. Various branches of the vagus nerve may be injured following an upper respiratory illness.^40^,^46^ Patients with this condition may present with breathy dysphonia, vocal fatigue, effortful phonation, odynophonia, cough, and/or dysphagia, lasting long after resolution of the acute viral illness.

These symptoms and findings are consistent with the hypothesis that, in London’s case, viral infection caused vagal neuritis complicated with recurrent laryngeal nerve dysfunction, vocal cord paralysis, and muscle atrophy.

As long as the left cord compensated and moved toward the impaired right vocal cord, sound was produced and London could continue singing. Because of his excellent technique and breath support, he was able to perform for a few more years. However, some high notes became increasingly difficult to sing, and he gradually lost vocal thrust. London’s gradual vocal deterioration described herein is highly suggestive of postviral recurrent laryngeal nerve palsy.

### Determining the Cause

Although the common belief was that George London’s career ended early due to vocal palsy caused by viral hepatitis, there is no evidence to support such an etiology. The negative findings on neck exploration eliminate the possible etiology of neoplasm. Vasculopathy might been a consideration; however, the long time between his first cardiovascular event and diagnosis of UVCP makes this quite unlikely. Idiopathic vocal cord paralysis may also be ruled out due to the course of London’s symptoms and no improvement over time. London’s young age, the diagnosis of laryngitis prior to his vocal palsy, and the course of his disease suggest that the underlying cause of the termination of his singing career was postviral vagal neuropathy.

## CONCLUSION

This paper has examined the unique case of a professional world-renowned opera singer and his efforts to prevent terminal dysfunction of his voice. The events that preceded his diagnosis, the efforts of experts in the field to treat him, and his brave battle with the consequences of his disease give a personal perspective on his condition. The description of vocal cord reconstruction attempts made by Dr Henry Rubin shed light on the development of this field during the last century. These early medical efforts and London’s courage in the face of his devastating condition set the stage for contemporary approaches and methods of in-office vocal cord augmentation injections using materials such as carboxymethylcellulose (Radiesse Voice Gel™), calcium hydroxylapatite (Radiesse™), and autologous fat.^53^

George London died peacefully in his sleep on Sunday evening March 24, 1985, at the age of 65, 24 years after being diagnosed with UVCP. London was one of the most impressive bass-baritones of the post-war era, with a distinctive voice and an imposing charismatic stage presence. His voice, artistry, and battle to continue singing serve as inspiration to future generations of singers and the doctors who treat them.

## Abbreviations

HSV: herpes simplex virus
UVCP: unilateral vocal cord palsy
